# Development and validation of nomogram based on miR-203 and clinicopathological characteristics predicting survival after neoadjuvant chemotherapy and surgery for patients with non-metastatic osteosarcoma

**DOI:** 10.18632/oncotarget.18534

**Published:** 2017-06-17

**Authors:** Dong Cheng, Xubin Qiu, Ming Zhuang, Chenlei Zhu, Hongjun Zou, Ailiang Zhang

**Affiliations:** ^1^ Department of Orthopedics, The Third Affiliated Hospital of Soochow University, Changzhou 213003, P.R. China

**Keywords:** microRNA-203, nomogram, prognosis, osteosarcoma, external validation

## Abstract

**Background:**

Recently, nomograms have been used as models for risk prediction in malignant tumor because they can predict the outcome of interest for a certain individual based on many variables. This study aimed to establish an effective prognostic nomogram for osteosarcoma based on the clinicopathological factors and microRNA-203.

**Results:**

The results showed that miR-203 expression was significantly lower in osteosarcoma tissues compared with the corresponding adjacent tissues (*P* < 0.001). Patients with low miR-203 expression had poor overall survival (OS) in osteosarcoma. The histological type, tumor size, AJCC stage and miR-203 expression were integrated in the nomogram. The nomogram showed significantly better prediction of OS than for patients with non-metastatic osteosarcoma. The ROC curve also showed higher specificity and sensitivity for predicting 3- and 5-year osteosarcoma patients’ survival compared with AJCC stage. The decision curve analysis also indicated more potential of clinical application of the nomogram compared with AJCC staging system. Moreover, our findings were supported by the validation cohort.

**Materials and Methods:**

We retrospectively investigated 301 patients with non-metastatic osteosarcoma. Data from primary cohort (*n* = 198) were used to develop multivariate nomograms. This nomogram was internally validated for discrimination and calibration with bootstrap samples and was externally validated with an independent patient cohort (*n* = 103).

**Conclusions:**

Our proposed nomogram showed more accurate prognostic prediction for patients with non-metastatic osteosarcoma.

## INTRODUCTION

Osteosarcoma is the most common primary malignant tumor of bone in children and adolescents with an incidence of 4–5 cases per million people [1]. With the development of its multidisciplinary treatment, the long-term survival of patients with osteosarcoma has improved dramatically [1]. Traditionally, the prognosis of osteosarcoma was performed according to the 7th edition American Joint Committee on Cancer tumor-node-metastasis (AJCC TNM) staging system or Enneking staging system. However, osteosarcoma patients at the same TNM stage or Enneking stage usually had variable outcomes, suggesting that the current staging system which only assesses tumor grade and size, skip metastases, and nodal or distant metastasis may be inadequate to make a treatment decision and evaluate the prognosis. In addition, with more and more clear understanding of the microRNA (miRNA) mechanisms in osteosarcoma pathogenesis, some miRNAs can be served as prognostic biomarkers [2]. Therefore, we need a new tool that can provide reliable prognostic information by incorporating multiple clinical variables and biomarkers. The visual format of nomogram is a simple and advanced prediction model that estimates the survival of individual patient by incorporating multiple variables [3]. The nomogram has been extensively used for many cancers, and it has been proposed as an alternative or even as a new standard [4–7].

MiRNAs are a class of endogenously expressed small non-coding RNAs with a length of 18–25 nucleotides. MiR-203 is originally known as a skin-specific miRNA and involved in regulating the embryonic epidermal differentiation [8]. Recently, it has been shown that miR-203 is a putative tumor suppressor and downregulated in various tumor. Since 2008, many studies have demonstrated the prognostic value of miR-203 in a variety of tumors. It has been reported that downregulated miR-203 expression was associated poor outcome in several types of cancers, including esophageal cancer [9], hepatocellular carcinoma [10], gastric cancer [11], lung cancer [12], and so on. In this study, we found that patients with decreased miR-203 expression had poor overall survival in osteosarcoma. MiR-203 is an independent prognostic biomarker. In addition, the prognostic nomogram for osteosarcoma based on the clinicopathological parameters and miR-203 provides more accurate prediction of patient survival compared with the TNM staging system.

## RESULTS

### miR-203 expression was decreased in osteosarcoma tissues

**Figure 1:**
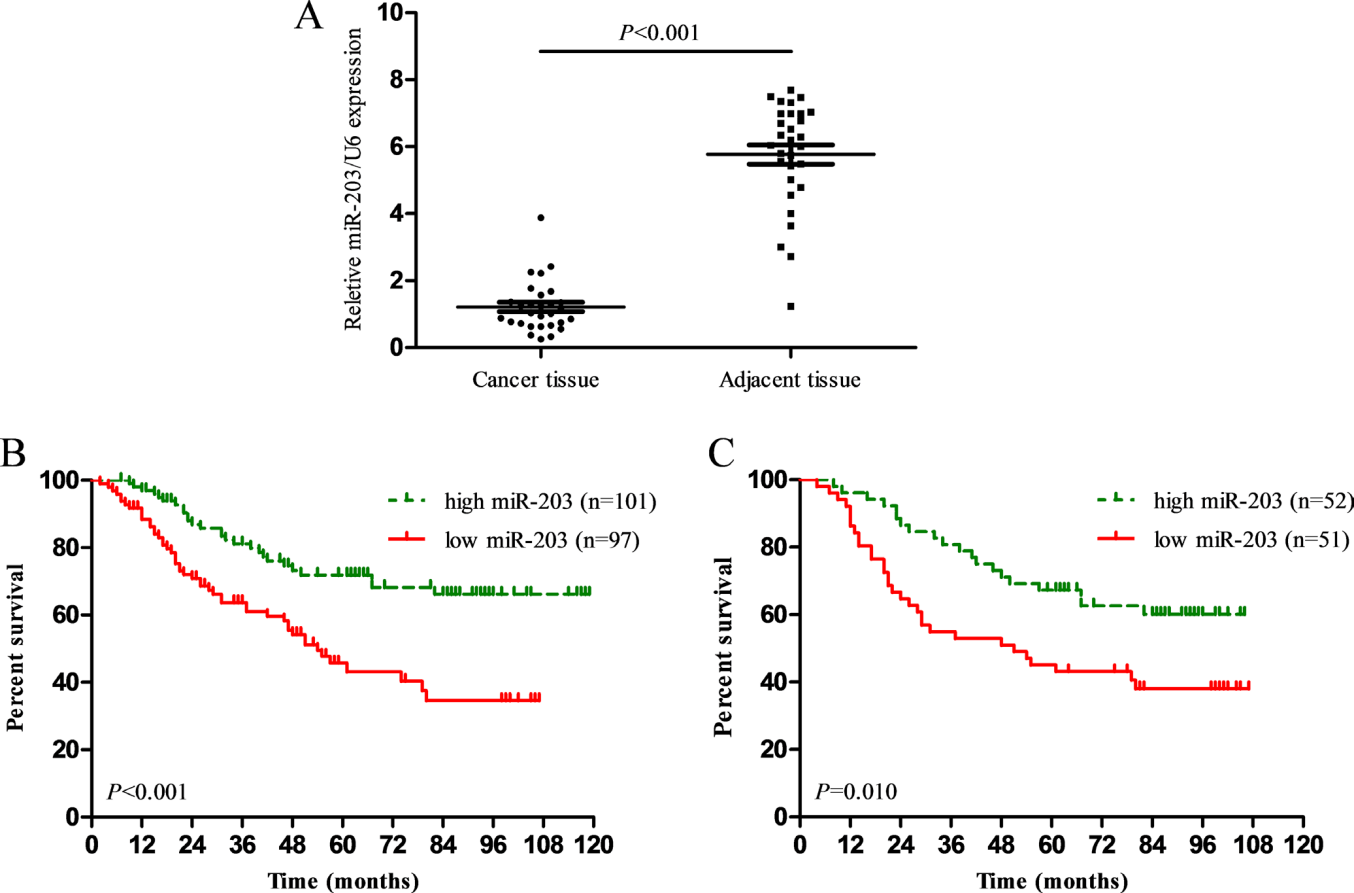
(**A**) The expression of miR-203 in 30 paired of osteosarcoma samples and their corresponding normal bone tissues was detected using qRT-PCR; (**B**) Kaplan-Meier curve for osteosarcoma patients classified as high or low miR-203 expression in primary cohort or (**C**) in validation cohort, the *P*-value was calculated using the log-rank test.

MiR-203 expression was analyzed by qRT-PCR. The relative expression of miR-203 in the cancer tissues normalized to U6 was 1.21 ± 0.23 (mean ± SD), while the relative expression of miR-203 in adjacent bone tissues was 5.76 ± 0.36. The result showed that miR-203 expression was significantly lower in osteosarcoma tissues compared with the corresponding adjacent bone tissues (*P* < 0.001, Figure 1A).

### Association between miR-203 expression and clinicopathological factors

The median miR-203 expression was used as a cutoff value to divide patients into high or low group. As shown in Table 1, we found that miR-203 expression was significantly associated with age (*P* = 0.026) and histological type (*P* = 0.002) in primary cohort. However, there were no significance between miR-203 expression and other clinicopathological factors, such as sex, histological grade, tumor size, and 7th AJCC stage in primary cohort. In validation cohort, miR-203 expression was not associated with any clinicopathological factors.

**Table 1:** Association of miR-203 expression with clinicopathological factors in osteosarcoma

Clinical parameter	Primary cohort				Validation cohort			
	MiR-203 High (101)	MiR-203 Low (97)	χ^2^	*P*	MiR-203 High (52)	MiR-203 Low (51)	χ^2^	*P*
Sex			0.50	0.481			1.20	0.274
Male	54	47			27	21		
Female	47	50			25	30		
Age			4.93	0.026^*^			1.18	0.277
< 20	49	64			28	22		
≥ 20	52	98			24	29		
Histological grade			3.14	0.370			0.99	0.805
Well differentiated	10	17			4	7		
Moderately differentiated	27	20			10	9		
Poorly differentiated	24	20			11	10		
Undifferentiated	40	40			27	25		
Histological type			16.67	0.002^*^			4.82	0.307
Osteoblastic	48	68			27	33		
Fibroblastic	25	8			14	7		
Chondroblastic	13	12			5	8		
Telangiectatic	7	1			3	1		
Others	8	8			3	2		
Tumor size			0.84	0.360			0.12	0.726
≤ 8 cm	45	37			18	16		
> 8 cm	56	60			34	35		
7th AJCC stage			2.43	0.297			0.51	0.775
I	37	36			14	16		
II	60	52			35	31		
III	4	9			3	4		

### Low miR-203 expression predicts poor survival in osteosarcoma patients

We evaluated the prognostic value of miR-203 expression in patients with osteosarcoma, by using the Kaplan-Meier survival analysis and log-rank test. As shown in Figure 1B and 1C, patients in low miR-203 expression group had poor overall survival (OS) compared with those in high miR-203 expression group in both primary cohort and validation cohort (*P* < 0.001, *P* = 0.010). Cox regression univariate analysis showed that age ≥ 20 years, undifferentiated, osteoblastic, tumor size > 8 cm, advanced stage, and low miR-203 expression were negative prognostic factors for OS in the primary cohort (*P* < 0.05, Table 2). However, sex had no effect on survival of patients (*P* > 0.05, Table 2). Cox regression multivariate analyses confirmed that histological type, tumor size, AJCC stage and miR-203 expression were independent prognostic factors (Table 2).

**Table 2:** Univariate and multivariate cox regression analyses for overall survival in patients with non-metastatic osteosarcoma

Variables	Univariate analysis		Multivariate analysis	
	HR (95% CI)	*P* value	HR (95% CI)	*P* value
Sex				
Male vs. Female	0.94 (0.60–1.48)	0.795		
Age				
≥ 20 years vs. < 20 years	1.58 (1.01–2.55)	0.048^*^	1.57 (0.94–2.64)	0.087
Histological grade		0.001^*^		0.08
Well differentiated	Ref.	–	Ref.	
Moderately differentiated	0.43 (0.14–1.32)	0.140	0.45 (0.17–1.21)	0.114
Poorly or not differentiated	1.82 (0.73–4.53)	0.198	0.89 (0.09–9.02)	0.920
Undifferentiated	2.23 (0.95–5.24)	0.065	0.49(0.05–4.93)	0.542
Histological type		< 0.001^*^		< 0.001^*^
Osteoblastic	Ref.		Ref.	
Fibroblastic	0.30 (0.14–0.67)	0.003	0.25 (0.10–0.60)	0.002
Chondroblastic	0.30 (0.13–0.70)	0.005	0.28 (0.12–0.65)	0.003
Telangiectatic	0.36 (0.08–1.46)	0.151	0.13 (0.03–0.61)	0.009
Others	0.10 (0.01–0.71)	0.021	0.08 (0.01–0.55)	0.011
Tumor size				
> 8 cm vs. ≤ 8 cm	1.71 (1.05–2.79)	0.031^*^	1.79 (1.02–3.17)	0.044^*^
7th AJCC stage		< 0.00^1*^		< 0.001^*^
I	Ref.		Ref.	
II	3.58 (1.88–6.82)	< 0.001	3.90 (0.39–39.29)	0.248
III	7.78 (3.10–19.47)	< 0.001	20.6 (2.26–187.7)	0.007
MiR-203				
Low vs. High	2.43 (1.52–3.90)	< 0.001^*^	1.81 (1.27–2.58)	0.001^*^

### Nomogram development and internal validation

**Figure 2:**
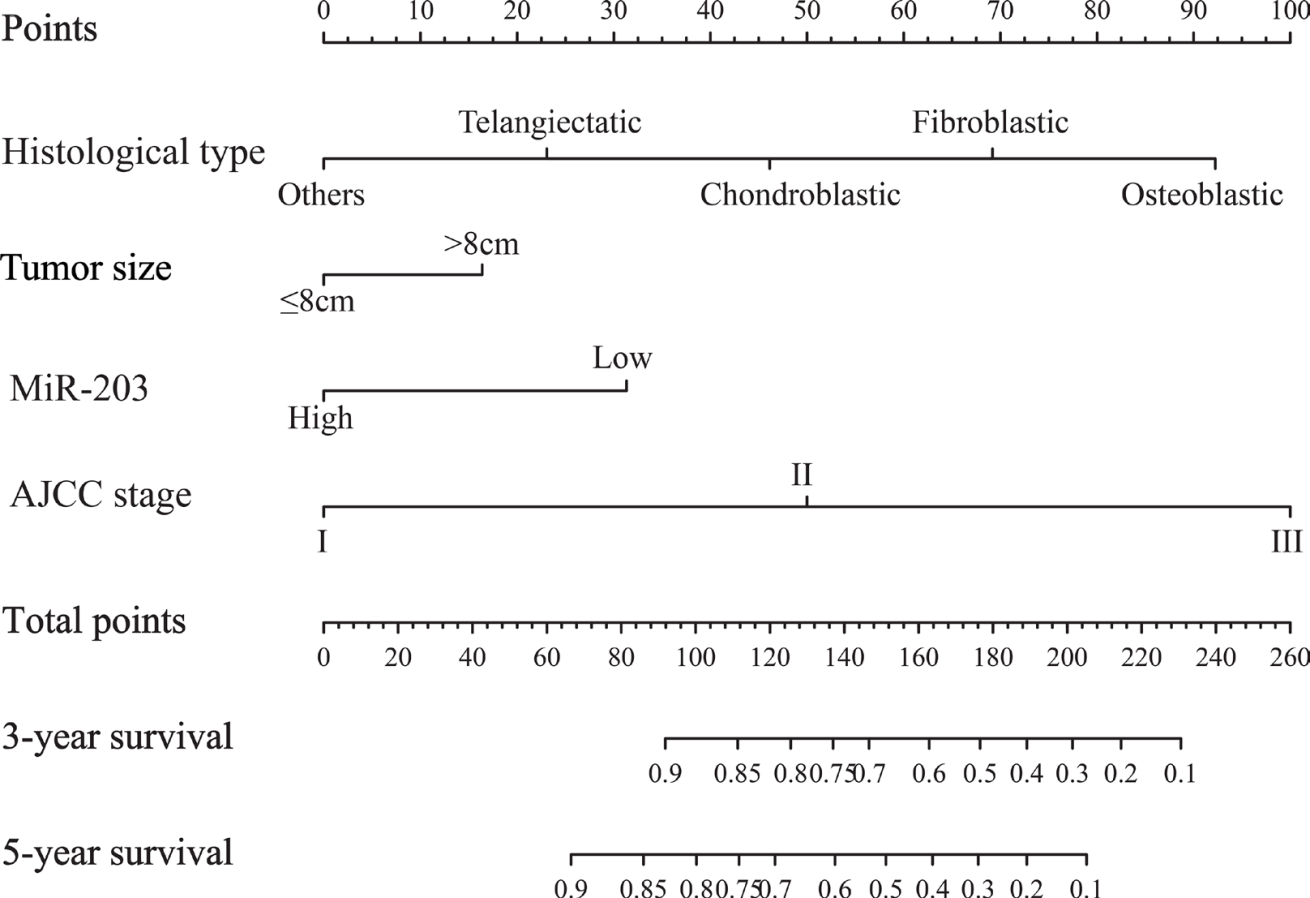
Evaluation of nomogram integrated miR-203 and clinicopathological factors in the patients with non-metastatic osteosarcoma To use the nomogram, the value attributed to an individual patient is located on each variable axis, and a line is drawn upwards to determine the number of points received for each variable value. The sum of these numbers is located on the total points axis, and a line is drawn downward to the survival axis to determine the likelihood of 3- or 5-year survival.

**Figure 3:**
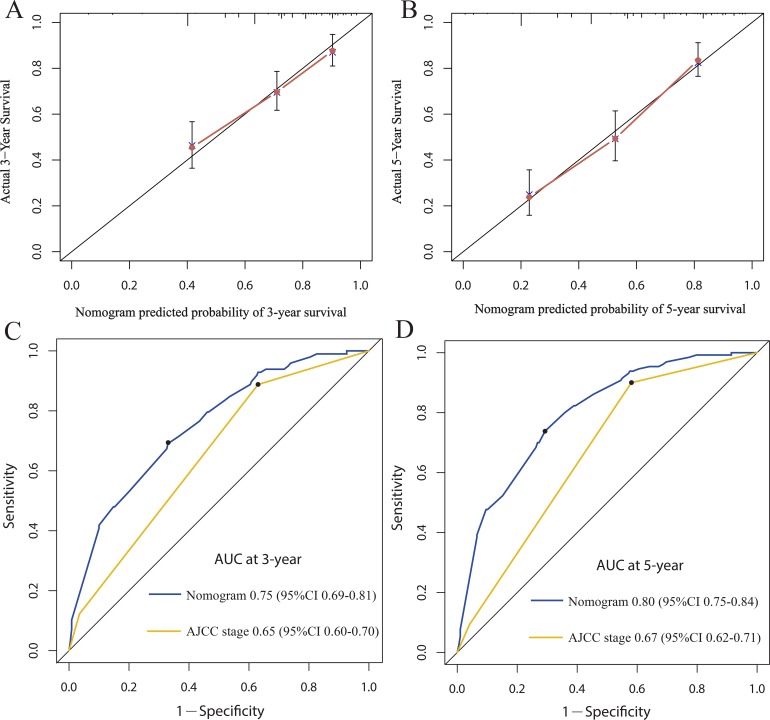
The calibration curve for predicting patient survival at 3-year (**A**) and 5-year (**B**) in the primary cohort. Time-dependent receiver operating characteristic (ROC) curves by nomogram and 7th AJCC-TNM staging system for 3-year (**C**) and 5-year (**D**) OS in the primary cohort.

**Figure 4:**
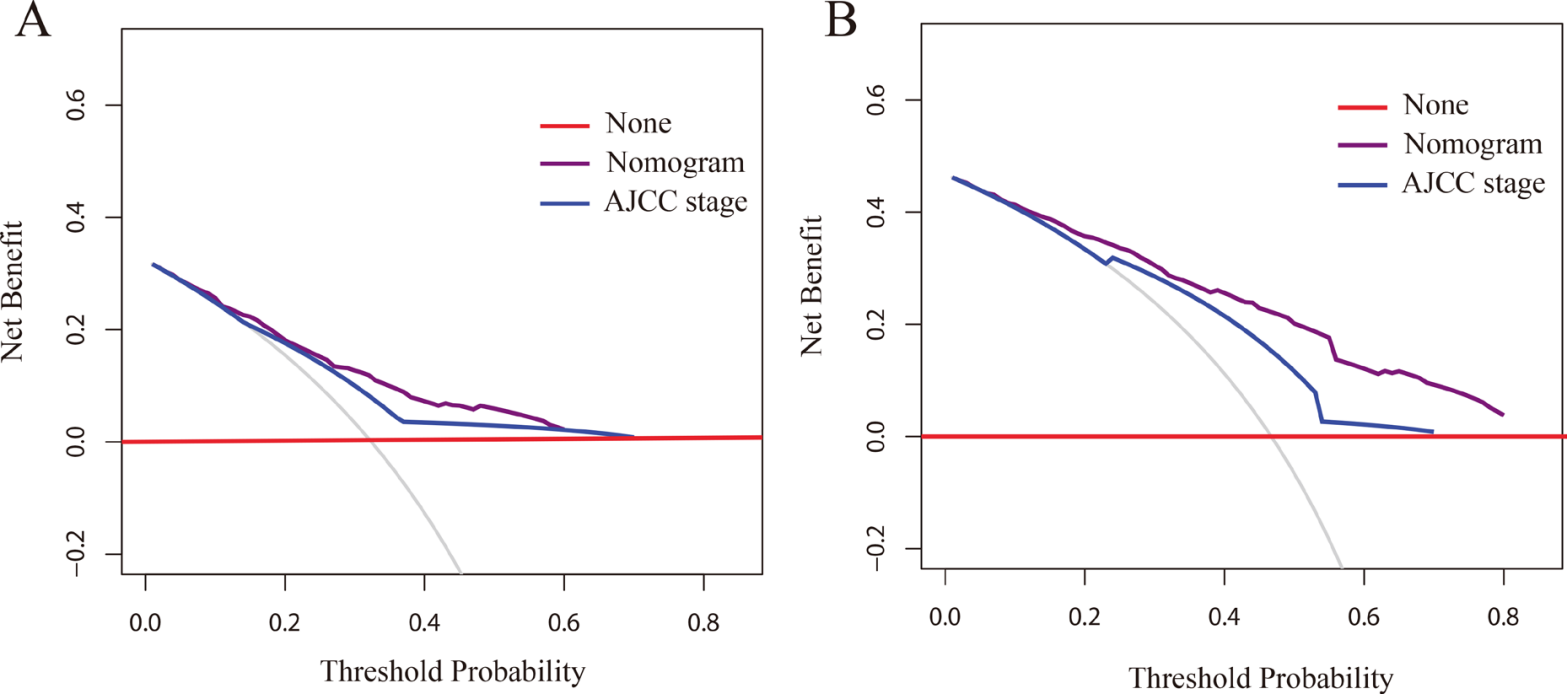
Decision curve analyses by nomogram and 7th AJCC-TNM staging system for 3-year (**A**) and 5-year (**B**) OS in the primary cohort.

For the development of the nomograms, patients’ data from Shanghai 6th Hospital were used. Multivariate analyses demonstrated that histological type, tumor size, AJCC stage and miR-203 expression were independent risk factors for OS in the primary cohort (Table 2). In order to find a best-fit model to evaluate osteosarcoma patients’ OS, a predictive nomogram was generated by backward stepwise selection with the AIC in Cox proportional hazards. Finally, the nomogram that integrated four variables: histological type, tumor size, AJCC stage and miR-203 expression was used to predict 3- and 5-year osteosarcoma patients’ survival (Figure 2). The calibration plot for the probability of survival at 3 or 5 years showed a perfect correlation between the actual observation and the prediction by the nomogram in primary cohort (Figure 2A and 2B). Our nomogram showed better accuracy for predicting osteosarcoma patients’ survival in the primary cohort. The C-index of the nomogram was 0.73 (95% CI 0.68–0.78), which was significantly higher than that of AJCC stage (C-index = 0.63, 95% CI 0.59–0.67) (*P* < 0.001). The calibration curves displayed good correlation between the actual observation and the prediction in the primary cohort (Figure 3A and 3B). The ROC curve displayed higher sensitivity and specificity for predicting osteosarcoma patients’ survival at 3- and 5-year (Figure 3C and 3D). In the decision curve analysis, the nomogram demonstrated high potential of clinical application because it ensured better net benefits throughout the entire range of threshold probabilities for survival after 3 or 5 years compared with AJCC stage (Figure 4A and 4B). These results indicated that our nomogram has better performance for predicting patients’ survival than AJCC staging system in osteosarcoma.

### External validation of the nomogram

**Figure 5:**
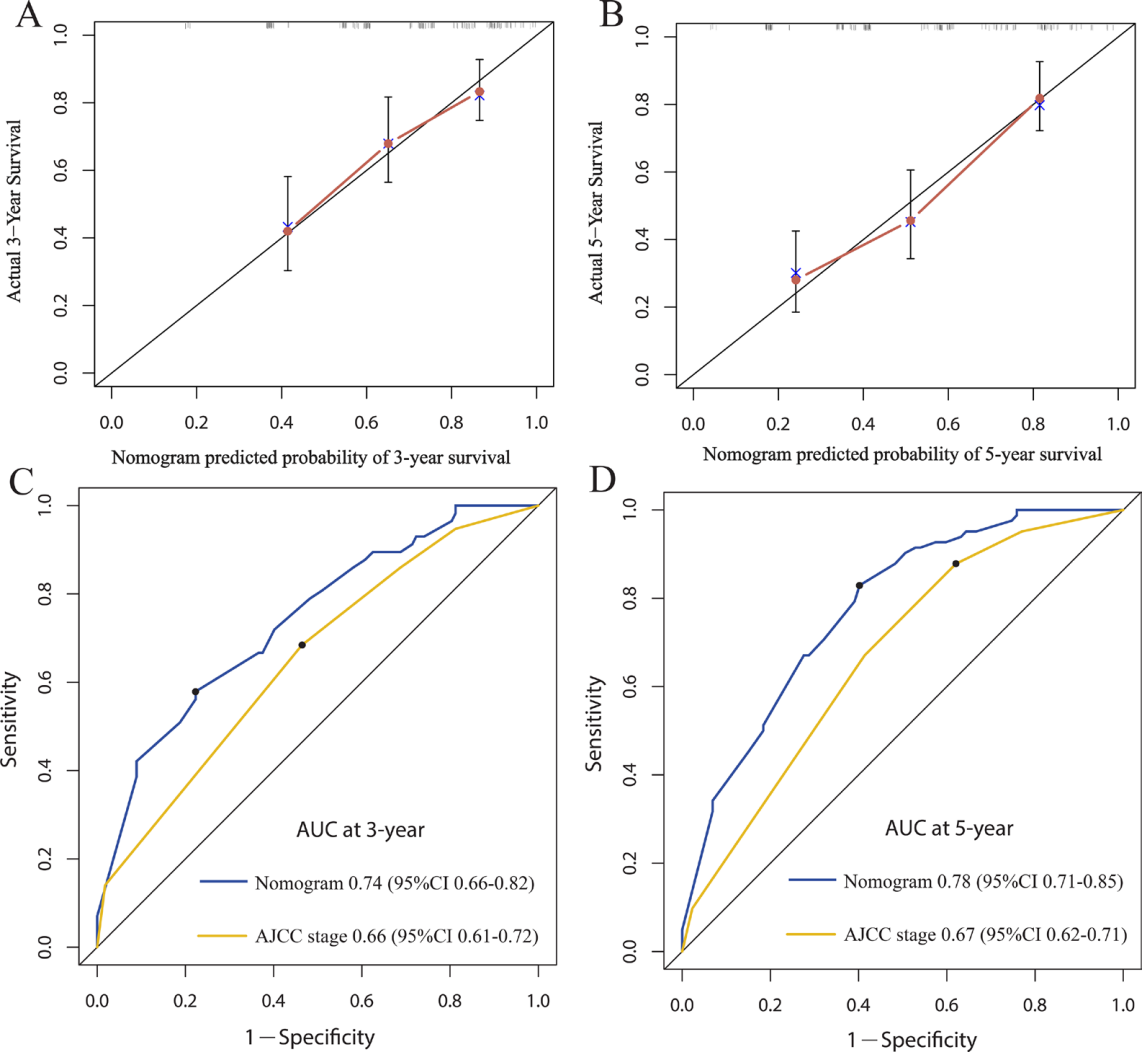
The calibration curve for predicting patient survival at 3-year (**A**) and 5-year (**B**) in the validation cohort. Time-dependent receiver operating characteristic (ROC) curves by nomogram and 7th AJCC-TNM staging system for 3-year (**C**) and 5-year (**D**) OS in the validation cohort.

The nomogram was externally validated in an independent validation cohort of 103 patients. The calibration curves displayed good correlation between the actual observation and the prediction in the validation cohort (Figure 5A and 5B). The C-index of the nomogram for predicting OS was 0.71 (95%CI 0.67 to 0.77) in the validation cohort, which was also significantly higher than AJCC stage (C-index = 0.62, 95% CI 0.57–0.68) (*P* < 0.001). The ROC curve displayed also higher sensitivity and specificity for predicting osteosarcoma patients’ survival at 3- and 5-year in the validation cohort (Figure 5C and 5D). These results indicated that the nomogram is an accurate and reliable tool for the predicting osteosarcoma patients’ survival.

## DISCUSSION

Recently, nomograms have been used as models for risk prediction in malignant tumor because they can predict the outcome of interest for a certain individual based on many variables [4–7]. Compare with AJCC TNM stage, nomograms have better performance for risk prediction. However, few data could be used to create predictive nomogram for osteosarcoma, because of its rarity and the difficulty in collecting enough patients. In our study. we developed and validated a prognostic nomogram that integrated not only clinicopathological features but also miR-203. The nomogram showed significantly better prediction of OS than for patients with non-metastatic osteosarcoma. The ROC curve also showed higher specificity and sensitivity for predicting 3- and 5-year osteosarcoma patients’ survival compared with AJCC stage. The decision curve analysis also indicated more potential of clinical application of the nomogram compared with AJCC staging system. Moreover, we performed the nomogram validation via the calibration plots and an independent external data set. The calibration plots showed perfect correlation between the predicted survival probability and the actual survival rate in both the primary and validation cohorts. The nomograms revealed an excellent correlation in predicting the survival probability in an independent validation cohort. Through internal and external validation, we believe our nomogram is a reliable and useful tool to predict survival in patients with non-metastatic osteosarcoma.

Until now, 4 prognostic nomograms for osteosarcoma have been available [13–16]. Kim et al. devised a nomogram for non-metastatic osteosarcoma that could predict risk of metastasis better AJCC staging system or tumor necrosis rate alone [15]. But it was designed without validation. Without validation, the result may not be relevant in other populations because of potential bias. Another nomogram showed the same problem [16]. Kim et al. have developed a new high-performance nomogram to predict the probability of metastasis in Enneking stage IIB extremity osteosarcoma and been validated in independent validation set [14]. However, the nomogram was based on 91 patients. The sample size is small. Ogura et al.’s nomogram was externally validated and verified to be useful for the prognosis for patients with non-metastatic osteosarcoma [13]. The nomogram was constructed following the necessary process of nomogram guideline. It was scientific and reliable. But the study did not compare the nomogram and AJCC stage which was better. In addition, the nomogram was constructed based on only clinicopathological factors. In our study, we developed a prognostic nomogram which integrated clinicopathological features and miR-203. It showed more accurately predict the prognosis of patients with non-metastatic osteosarcoma than AJCC stage.

MiR-203 is located in a frequently lost chromosomal region in T cell malignancies [17], and its expression is downregulated in tumors [9–12, 18, 19]. Previous study has demonstrated miR-203 was a putative tumor suppressor gene in several tumors. In prostate cancer, overexpression miR-203 controls proliferation, migration and invasion by directly targeting ZEB2, Bmi-1, survivin and LASP1 [20, 21]. In bladder cancer, decreased miR-203 predicts progression and poor prognosis for BC patients treated with cisplatin-based chemotherapy, and miR-203 overexpression can enhance cisplatin sensitization by promoting apoptosis via directly targeting Bcl-w and surviving [22]. In leukemia stem cells, down-regulation of miR-203 increases proliferation and self-renewal capacities via targeting of survivin and Bmi-1 [23]. However, overexpression miR-203 showed poor prognosis and may also exhibit oncogenic potential in colorectal cancer and pancreatic cancer [24–26]. Thus, miR-203 acts as a tumor suppressor or an oncogene based on tumor type. Recently, Liu et al. reported miR-203 was down regulated in osteosarcoma tissues and cell lines. Decreased miR-203 was associated with a poor prognosis in osteosarcoma patients [18]. Inhibition of miR-203 stimulated osteosarcoma cell growth by targeting TBK1, proliferation and RAB22A [18, 27, 28]. In current study, we found miR-203 expression was significantly lower in osteosarcoma tissues compared with the corresponding adjacent tissues. Patients with low miR-203 expression had poor OS compared with those with high miR-203 expression in both primary and validation cohorts. MiR-203 expression was an independent prognostic factor in osteosarcoma. So, miR-203 act as a tumor suppressor in osteosarcoma, and its expression including in our nomogram is reliable.

Although our nomogram is scientific and reliable, there are several limitations to the current study. First, we only include miR-203 in the nomogram. The inclusion of other miRNA such as miR-21, miR-214 and miR-9 would enhance the predictive ability of future nomograms; Second, it was performed with retrospective data, and there may have resulted in selection bias during data collection. Third, we used the surgically resected osteosarcoma tumor tissues and the adjacent tissues. Then, this nomogram would be applied only after surgical treatment. Thus, there is no clinical usefulness of this nomogram at the diagnosis, since the standard treatment of osteosarcoma includes neoadjuvant chemotherapy including methotrexate, cisplatin, and doxorubicin. Fourth, All of the subjects we selected received neoadjuvant chemotherapy combined with surgery, but there were several different neoadjuvant chemotherapy regimens. Different chemotherapy may have different effects on miR-203 expression, which require further study to confirm. In future studies, we will expand the sample size and choose patients who receive the same neoadjuvant chemotherapy in order to eliminate the interference of confounding factors and obtain more accurate clinical significance of miR-203. In addition, the miRNA profiling of the resected tumor tissues must be very likely modified by the neoadjuvant chemotherapy. In further study, we need compare the miR-203 levels between the biopsy specimens and the resected tumor tissues, and clarify that miR-203 levels are affected or not by the chemotherapeutic agents using *in vitro* and *vivo* experimental procedures. Finally, although our nomogram was reliable using external validation, it is necessary to verify by further external validation using a cross-racial patient cohort.

In conclusion, our proposed nomogram integrated clinicopathological factors and miR-203 can accurately predict the prognosis of patients with non-metastatic osteosarcoma. We believe that our nomogram is a reliable and useful tool, which can facilitate making the therapeutic decision and individualized patient counseling.

## MATERIALS AND METHODS

### Patients

A total of 198 osteosarcoma patients who underwent radical surgery at the Shanghai Hospital between January 2002 and December 2011 were enrolled in this study according to the following criteria: 1) histological diagnosis of osteosarcoma, 2) no distant metastasis at presentation, 3) R0 resection, 4) undergone standard therapy (neoadjuvant chemotherapy, and definitive surgery), 5) follow-up period of more than 3 years for survivors. In addition, 103 patients from the Third Affiliated Hospital of Soochow University were enrolled in the validation cohort of this study between January 2002 and December 2011. This study was approved by the Third Affiliated Hospital of Soochow University according to the Declaration of Helsinki. Written informed consent was obtained from all participants.

### Quantitative reverse transcription-polymerase chain reaction analysis (qRT-PCR)

30 paired fresh surgically resected osteosarcoma tumor tissues and adjacent non-tumor bone tissues were collected from Third Affiliated Hospital of Soochow University between January 2012 and December 2016. The specimens were immediately frozen in liquid nitrogen and stored at −80°C until use. Total RNA was extracted from tissues and cells using miRNeasy Mini Kit (QIAGENE, Shanghai, China), according to the manufacturer’s protocol. Total RNA concentration was assessed by measuring absorbance at 260 nm using a NanoDrop spectrophotometer (ND-1000, Thermo Scientific, Waltham, MA, USA). 2 μg of total RNA was reversely transcribed using the PrimeScript RT reagent kit with gDNA Eraser (TaKaRa,Japan) and miRNA-specific stem-loop RT primer (Applied Biosystems, USA). Stem-loop RT primer for miR-203 was: 5′- GTC GTA TCC AGT GCA GGG TCC GAG GTA TTC GCA CTG GAT ACG ACC TAG TGGTC-3′. Genespecific amplification was performed using ABI 7500 fast real-time PCR system (Applied Biosystems, Foster City, CA, USA) and SYBR Green PCR Master Mix (Applied Biosystems, Foster City, CA, USA) according to the manufacturer’s instructions. The following gene-specific primers were used in this study: forward, 5′-GGGGTGAAATGTTTAGGA-3′ and reverse 5′-GTGCGTGTCGTGGAGTCG-3′ for miR-203; forward, 5′-AGCCATATCTCCCACCCTGA-3′ and reverse 5′- TGGTGTGGCTTTAGTGCTCC -3′ for U6. The relative expression level of miR-203 were normalized to that of internal control U6 using the comparative delta CT (2^–ΔΔCt^) method.

### Statistical analysis

The expression of miR-203 between osteosarcoma tissues and adjacent tissues was compared with *T* test. Survival curves were made using the Kaplan-Meier method and compared using the log-rank test. Nomogram construction and validation were performed with Iasonos’ guide [29]. The analysis of time-dependent receiver operating characteristics (ROC) curve and concordance index (C-index) were used to compare the discrimination power for OS between different models. Confidence intervals (CIs) were obtained by creating 500 bootstrap samples from the entire dataset and replicating the estimation process. The larger the C-index, the more accurate was the prognostic prediction. Statistical analysis was carried out using SPSS 21.0 for windows (SPSS, Chicago, IL), Graphpad Prism 5 (La Jolla, CA, USA) and R software version 3.2.5 (http://www.r-project.org/) with rms, Hmisc and survival ROC packages. A *P* value less than 0.05 was considered to be statistically significant unless otherwise specified.
